# Supplementary material for: Overall and cause-specific hospitalisation and death after COVID-19 hospitalisation in England: A cohort study using linked primary care, secondary care, and death registration data in the OpenSAFELY platform
Source: PLoS Med. 2022 Jan 25;19(1):e1003871. doi: 10.1371/journal.pmed.1003871 (PMC8789178; doi:10.1371/journal.pmed.1003871)
Supplement: S1 Outline Study Plan — (PDF) [file pmed.1003871.s001.pdf]

<u>ORIGINAL OUTLINE STUDY PLAN</u> (as documented on 12th February 2021)

Title: Readmissions among those discharged following a COVID-19 hospitalisation

Study type: Descriptive

Aim: To describe the rate of readmissions after a first COVID-19 hospitalisation, and reasons for re-admission. To aid interpretation, comparisons with those who have not been hospitalised with COVID-19 will also be made.

Setting: OpenSAFELY data based on TPP primary care, with linkage to SUS hospitalisations data and ONS mortality data.

Study population:
People with a first hospitalisation for COVID-19 (defined as code U071 or U072 as the primary reason for hospitalisation - in a secondary analysis one of these codes anywhere in the hospitalisation will be counted).

Comparison group:
People in the main study cohort will be matched to people in the general population who have not had a COVID-19 hospitalisation, for comparison. Each COVID-19 hospitalised patient will be matched to up to 10 people who were under follow-up in TPP and not hospitalised on the 1st day of the month of hospitalisation of the index case (the 1st day of the month rather than exact day is chosen due to computational limitations). Matching will be on age (nearest age, with a maximum window of 3 years), sex, and STP (NHS administrative region).

Outcome
The outcome is hospital (re)admission.

Analysis plan:
The demographic characteristics and comorbidity profile of patients hospitalised for COVID-19 and their matched controls will be described.

A time-to-event analysis will be done, with follow-up starting on date of initial discharge (and 1st of the same month, for controls). Cumulative incidence of COVID-19 (re)admission, non-COVID hospitalisation and death will be calculated and displayed, allowing for competing risks.

ICD-10 codes denoting the reason for (re)admission will be categorised into broad groups (following Bhaskaran et al doi.org/10.1016/S2213-8587(18)30288-2) and described.

Subdistribution hazard ratios for reason-specific hospitalisation comparing COVID-19 discharged patients with controls will be estimated using Fine and Gray models, initially accounting for matching factors only, and then for with adjustment for comorbidities.

Multiple imputation will be used to handle missing ethnicity. Missing BMI will be assumed healthy, and missing smoking status will be assumed to mean non-smoker, since these variables are unlikely to be missing at random.

Changes from the outline plan, and justification

The outline above was set out at an early stage in the design process, and some design decisions were changed during the course of planning the study in detail. Changes (1) to (5) below were made prior to any data analysis. Change (6) was made after initial descriptive analysis.

1. It was decided to use a general population comparison group from the 2019 population rather than a contemporary comparator, because it became clear from evidence outside this study that consultation for and recording of non-COVID health outcomes was highly atypical during the pandemic period, which would have made results difficult to interpret.

2. The matching ratio was reduced from 10:1 to 5:1 to ensure a high matching success rate and reduce computation time.

3. It was decided to add a second comparison group comprising patients hospitalised with influenza in previous years, to account for risks associated with hospitalisation in general, and to provide a benchmark against a well-known and established infectious disease. This was informed by similar approaches being developed in other OpenSAFELY projects.

4. Death was included in the overall hospitalisation outcome (making a composite of hospitalisation and death) to ensure that we fully captured serious post-COVID adverse outcomes in a public health-relevant way. All-cause mortality was added as an additional outcome to provide additional insight. Similarly cause-specific deaths were included with cause-specific hospitalisation outcomes.

5. Modelling of cause-specific hazards was used in the primary analysis of cause-specific outcomes, to prioritise estimation of associations among people “still at risk” of the outcome at any given moment, which we judged to be of primary interest; the originally planned Fine and Gray models were also fitted in a secondary analysis.

6. It was decided to exclude the first week of follow-up after discharge because early descriptive data showed a high rate of readmissions and deaths in the first week (i.e. very close to, or indeed part of the initial acute episode of COVID-19), which would have dominated most of the results, making the findings less informative for our main aim of describing outcomes after the initial acute illness.
