# Supplementary material for: Overall and cause-specific hospitalisation and death after COVID-19 hospitalisation in England: A cohort study using linked primary care, secondary care, and death registration data in the OpenSAFELY platform
Source: PLoS Med. 2022 Jan 25;19(1):e1003871. doi: 10.1371/journal.pmed.1003871 (PMC8789178; doi:10.1371/journal.pmed.1003871)
Supplement: S1 IG Statement — (PDF) [file pmed.1003871.s002.pdf]

Information Governance Statement

NHS England is the data controller; TPP is the data processor; and the key researchers on OpenSAFELY are acting on behalf of NHS England. OpenSAFELY is hosted within the TPP environment which is accredited to the ISO 27001 information security standard and is NHS IG Toolkit compliant [1,2]; patient data are pseudonymised for analysis and linkage using industry standard cryptographic hashing techniques; all pseudonymised datasets transmitted for linkage onto OpenSAFELY are encrypted; access to the platform is via a virtual private network (VPN) connection, restricted to a small group of researchers who hold contracts with NHS England and only access the platform to initiate database queries and statistical models. All database activity is logged. No patient-level data leave the platform; only aggregate statistical outputs leave the platform environment following best practice for anonymisation of results such as statistical disclosure control for low cell counts [3]. The OpenSAFELY platform adheres to the data protection principles of the UK Data Protection Act 2018 and the EU General Data Protection Regulation (GDPR) 2016. In March 2020, the Secretary of State for Health and Social Care used powers under the UK Health Service (Control of Patient Information) Regulations 2002 (COPI) to require organisations to process confidential patient information for the purposes of protecting public health, providing healthcare services to the public and monitoring and managing the COVID-19 outbreak and incidents of exposure [4]. Taken together, these provide the legal bases to link patient datasets on the OpenSAFELY platform. The study was approved by the Health Research Authority (REC reference 20/LO/0651) and by the LSHTM Ethics Board (ref 21863).

References for Information Governance Statement

1. NHS Digital. BETA – Data Security Standards 2020 [cited 2020 6th August]. Available from: https://digital.nhs.uk/about-nhs-digital/our-work/nhs-digital-data-and-technology-standards/framework/beta---data-security-standards.

2. NHS Digital. Data Security and Protection Toolkit. 2020 [cited 2020 6th August]. Available from: https://digital.nhs.uk/data-and-information/looking-after-information/data-security-and-information-governance/data-security-and-protection-toolkit.

3. NHS Digital. ISB1523: Anonymisation Standard for Publishing Health and Social Care Data 2020 [cited 2020 6th August]. Available from: https://digital.nhs.uk/data-and-information/information-standards/information-standards-and-data-collections-including-extractions/publications-and-notifications/standards-and-collections/isb1523-anonymisation-standard-for-publishing-health-and-social-care-data.

4. Secretary of State for Health - UK Government. Coronavirus (COVID-19): notification to organisations to share information 2020 [cited 2020 6th August]. Available from: https://www.gov.uk/government/publications/coronavirus-covid-19-notification-of-data-controllers-to-share-information.
