# Supplementary material for: Overall and cause-specific hospitalisation and death after COVID-19 hospitalisation in England: A cohort study using linked primary care, secondary care, and death registration data in the OpenSAFELY platform
Source: PLoS Med. 2022 Jan 25;19(1):e1003871. doi: 10.1371/journal.pmed.1003871 (PMC8789178; doi:10.1371/journal.pmed.1003871)

Accompanies *Bhaskaran et al. Overall and cause-specific hospitalisation and death after COVID-19 hospitalisation in England: a cohort study using linked primary care, secondary care and death registration data in the OpenSAFELY platform.*

S1 Figure: Distribution of entry dates for those in the COVID-19 hospitalised group, and the influenza-hospitalised and matched general population comparison groups

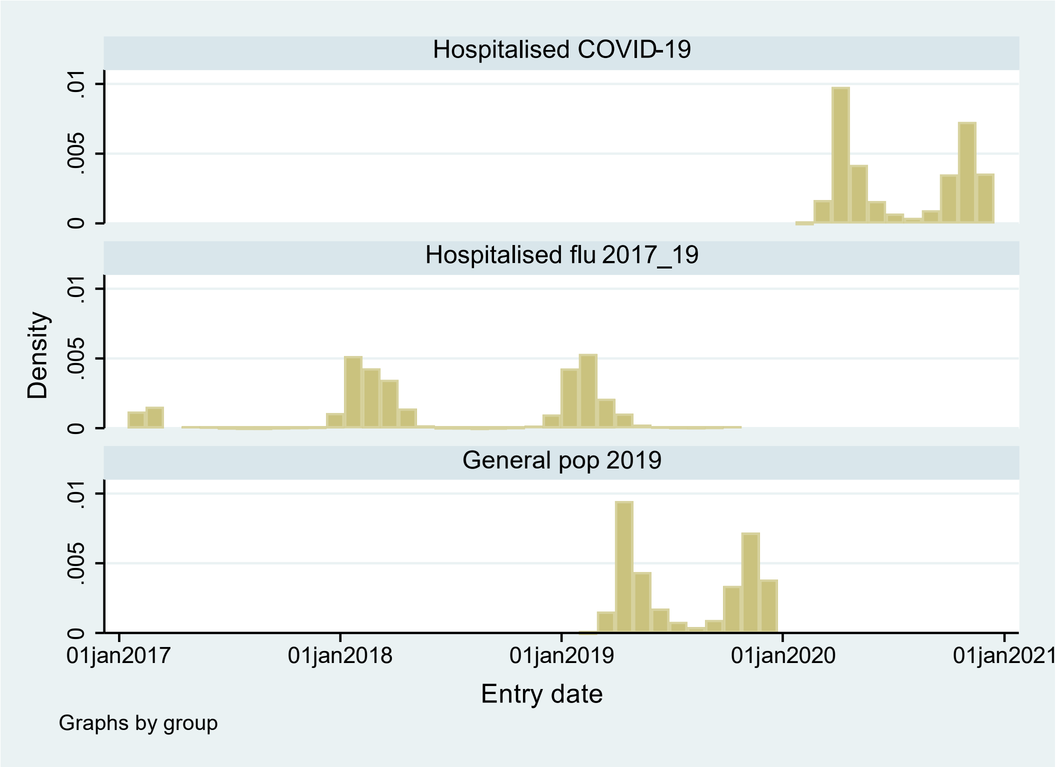

Supplement: S1 Fig — COVID-19, Coronavirus Disease 2019. (PDF) [file pmed.1003871.s004.pdf]
