# Supplementary material for: Overall and cause-specific hospitalisation and death after COVID-19 hospitalisation in England: A cohort study using linked primary care, secondary care, and death registration data in the OpenSAFELY platform
Source: PLoS Med. 2022 Jan 25;19(1):e1003871. doi: 10.1371/journal.pmed.1003871 (PMC8789178; doi:10.1371/journal.pmed.1003871)
Supplement: S2 Fig — COVID-19, Coronavirus Disease 2019; HR, hazard ratio; LRTI, lower respiratory tract infection. (PDF) [file pmed.1003871.s005.pdf]

Accompanies *Bhaskaran et al. Overall and cause-specific hospitalisation and death after COVID-19 hospitalisation in England: a cohort study using linked primary care, secondary care and death registration data in the OpenSAFELY platform.*

S2 Figure: Changes over time in the hazard ratios comparing outcomes in the COVID-19 and control groups

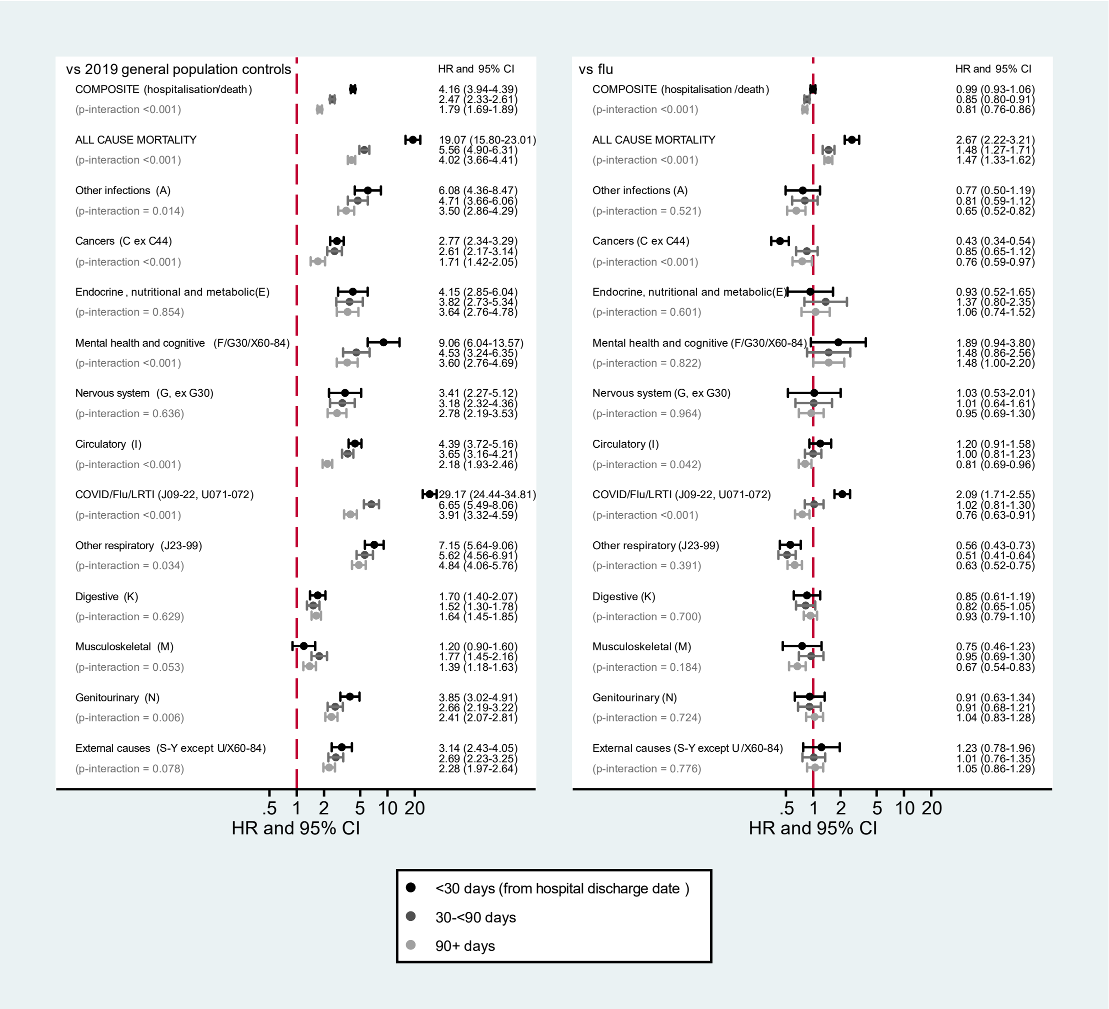

Note: hazard ratios from models containing an interaction between exposure and follow-up time, adjusted for age, sex and geography only
