# Supplementary material for: Overall and cause-specific hospitalisation and death after COVID-19 hospitalisation in England: A cohort study using linked primary care, secondary care, and death registration data in the OpenSAFELY platform
Source: PLoS Med. 2022 Jan 25;19(1):e1003871. doi: 10.1371/journal.pmed.1003871 (PMC8789178; doi:10.1371/journal.pmed.1003871)
Supplement: S3 Fig — aHR, adjusted hazard ratio; COVID-19, Coronavirus Disease 2019; HR, hazard ratio; sHR, subdistribution hazard ratio. (PDF) [file pmed.1003871.s006.pdf]

Accompanies *Bhaskaran et al. Overall and cause-specific hospitalisation and death after COVID-19 hospitalisation in England: a cohort study using linked primary care, secondary care and death registration data in the OpenSAFELY platform.*

S3 Figure: Adjusted hazard ratios/subdistribution hazard ratios in sensitivity analyses

(a) Comparisons with influenza controls

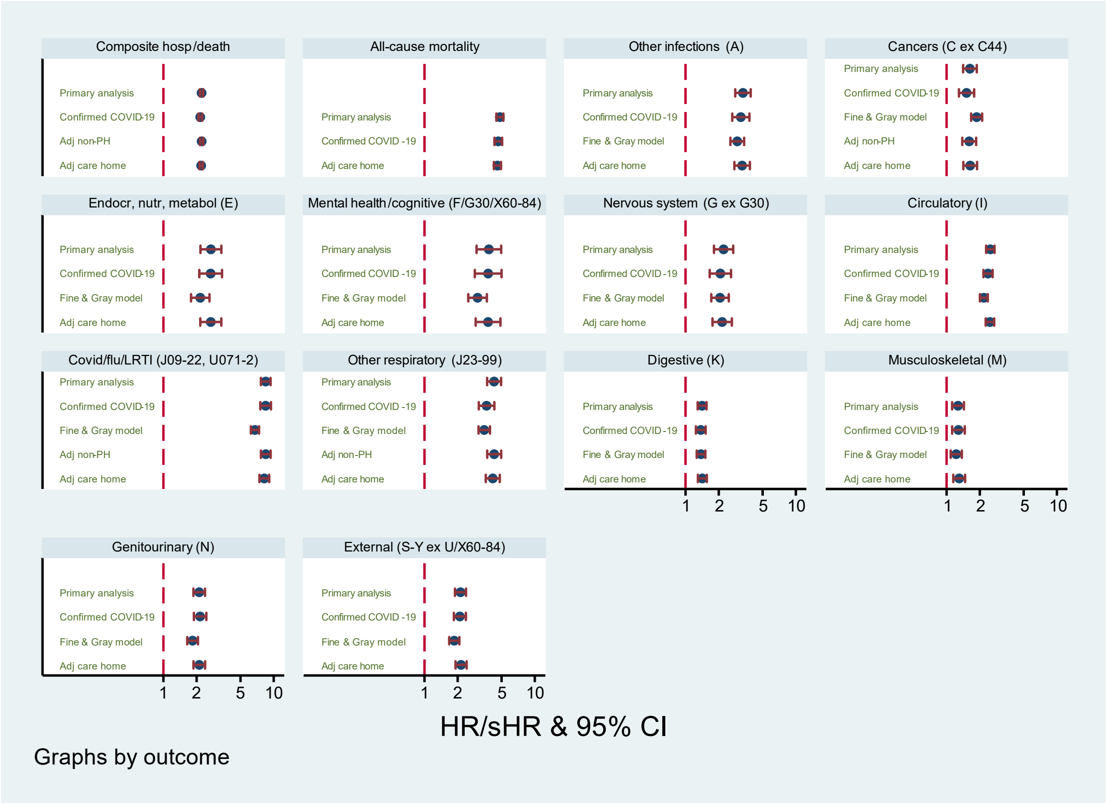

(b) Comparisons with influenza controls

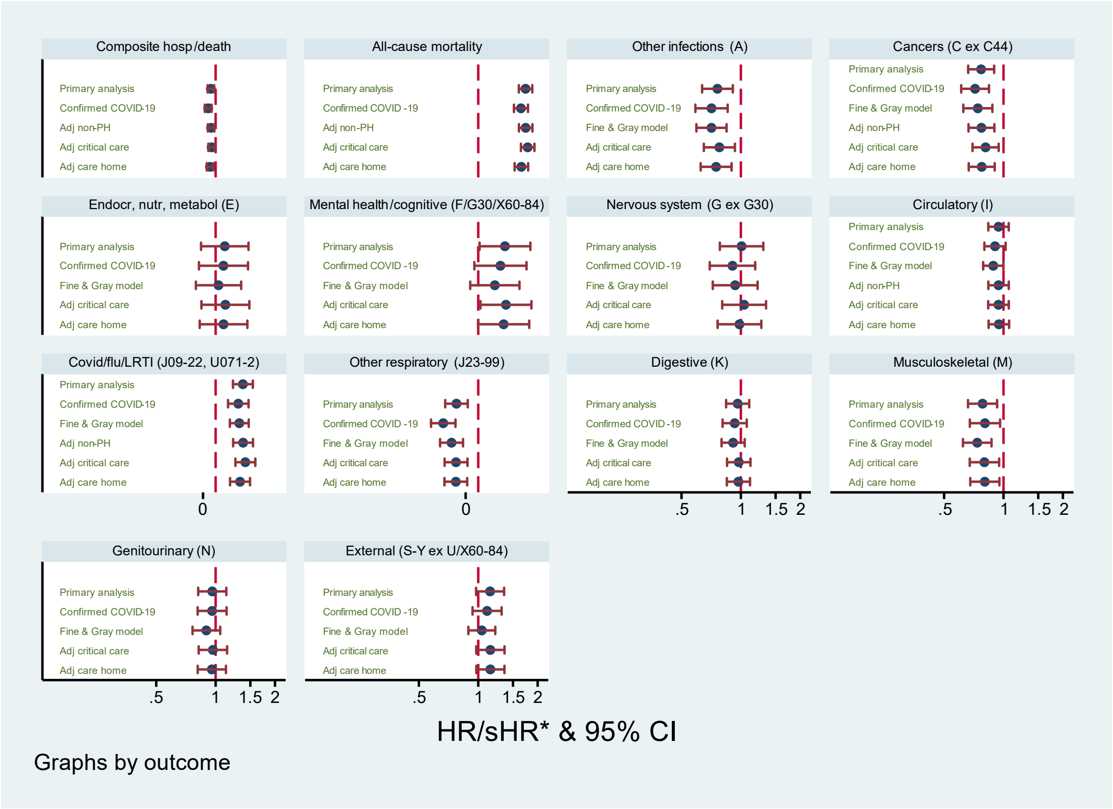

Notes: all models are fully adjusted for demographic and lifestyle-related characteristics and comorbidities (see methods). "Confirmed COVID-19" restricted the COVID-19 group to those with the ICD-10 code "U07.1 (COVID-19 – virus confirmed)" as the primary reason for hospitalisation. "Fine & Gray model" used Fine and Gray competing risks regression in place of Cox regression. "Adj non-PH" included interactions between follow-up time and adjustment covariates where proportional hazards checks had indicated evidence of non-proportionality. "Adj critical care" added adjustment for a binary variable indicating whether initial hospitalisation included critical care (for COVID-19 vs influenza comparison only). "Adj care home" added adjustment for a binary covariate indicating residence in a care home.
