# Supplementary material for: Overall and cause-specific hospitalisation and death after COVID-19 hospitalisation in England: A cohort study using linked primary care, secondary care, and death registration data in the OpenSAFELY platform
Source: PLoS Med. 2022 Jan 25;19(1):e1003871. doi: 10.1371/journal.pmed.1003871 (PMC8789178; doi:10.1371/journal.pmed.1003871)
Supplement: S1 Table — (PDF) [file pmed.1003871.s007.pdf]

Accompanies *Bhaskaran et al. Overall and cause-specific hospitalisation and death after COVID-19 hospitalisation in England: a cohort study using linked primary care, secondary care and death registration data in the OpenSAFELY platform.*

S1 Table: Demographic characteristics of people excluded from complete case analyses due to missing obesity or smoking data

		**Hospitalised with COVID-19**		**Matched controls from 2019 general population**		**Hospitalised with influenza in 2017-19**	
		**Missing data**	**No missing data**	**Missing data**	**No missing data**	**Missing data**	**No missing data**
**N (%)**		1520 (100.0)	23153 (100.0)	9605 (100.0)	113757 (100.0)	1154 (100.0)	14904 (100.0)
**Age (yrs)**	18-39	393 (25.9)	1642 (7.1)	2384 (24.8)	7791 (6.8)	404 (35.0)	1620 (10.9)
	40-49	252 (16.6)	2504 (10.8)	1543 (16.1)	12237 (10.8)	136 (11.8)	1326 (8.9)
	50-59	321 (21.1)	4358 (18.8)	1995 (20.8)	21400 (18.8)	155 (13.4)	1971 (13.2)
	60-69	203 (13.4)	4399 (19.0)	1382 (14.4)	21628 (19.0)	133 (11.5)	2520 (16.9)
	70-79	154 (10.1)	4880 (21.1)	991 (10.3)	24179 (21.3)	126 (10.9)	3366 (22.6)
	80+	197 (13.0)	5370 (23.2)	1310 (13.6)	26522 (23.3)	200 (17.3)	4101 (27.5)
	Median (IQR)	53 (39-67)	66 (54-79)	54 (40-69)	67 (54-79)	53 (32-72)	70 (54-81)
**Sex**	Male	835 (54.9)	12898 (55.7)	6509 (67.8)	62153 (54.6)	593 (51.4)	6504 (43.6)
	Female	685 (45.1)	10255 (44.3)	3096 (32.2)	51604 (45.4)	561 (48.6)	8400 (56.4)
**Index of Multiple Deprivation**	1 (least deprived)	271 (17.8)	4351 (18.8)	1647 (17.1)	23781 (20.9)	214 (18.5)	3068 (20.6)
	2	290 (19.1)	4453 (19.2)	1811 (18.9)	23448 (20.6)	221 (19.2)	3030 (20.3)
	3	271 (17.8)	4407 (19.0)	1821 (19.0)	21682 (19.1)	239 (20.7)	3033 (20.4)
	4	331 (21.8)	4852 (21.0)	2016 (21.0)	22206 (19.5)	217 (18.8)	2916 (19.6)
	5 (most deprived)	357 (23.5)	5090 (22.0)	2310 (24.0)	22640 (19.9)	263 (22.8)	2857 (19.2)

Note: Missing data columns represent individuals with missing body mass index or smoking. No missing data columns represent individuals with complete data on both of these items.
