# Supplementary material for: Overall and cause-specific hospitalisation and death after COVID-19 hospitalisation in England: A cohort study using linked primary care, secondary care, and death registration data in the OpenSAFELY platform
Source: PLoS Med. 2022 Jan 25;19(1):e1003871. doi: 10.1371/journal.pmed.1003871 (PMC8789178; doi:10.1371/journal.pmed.1003871)
Supplement: S2 Table — (PDF) [file pmed.1003871.s008.pdf]

Accompanies *Bhaskaran et al. Overall and cause-specific hospitalisation and death after COVID-19 hospitalisation in England: a cohort study using linked primary care, secondary care and death registration data in the OpenSAFELY platform.*

S2 Table: Distribution of first outcomes (hospital admission or death) among included individuals

	**Hospitalised with COVID-19**	**Hospitalised with influenza in 2017-19***	**Matched controls from 2019 general population**
N	24673	16058 (6689 in 2019*)	123362
Any hospitalisation or death (composite outcome)	6499 (26.3)	7788 (48.5)	16681 (13.5)
Deaths	2022 (8.2)	1513 (9.4)	3904 (3.2)
**Cause-specific admissions/deaths total [deaths] (%)**			
Other infections (ICD-10 codes beginning A)	391 [<=5] (1.6)	255 [<=5] (3.8)	569 [6] (0.5)
Cancers (C, ex C44)	566 [174] (2.3)	355 [60] (5.3)	1478 [242] (1.2)
Endocrine, nutritional and metabolic (E)	218 [29] (0.9)	98 [13] (1.5)	361 [28] (0.3)
Mental health and cognitive (F/G30/X60-84)	248 [111] (1.0)	68 [26] (1.0)	356 [149] (0.3)
Nervous system (G, ex G30)	240 [84] (1.0)	116 [21] (1.7)	467 [98] (0.4)
Circulatory (I)	1039 [180] (4.2)	511 [56] (7.6)	2256 [300] (1.8)
COVID-19/Influenza/LRTI (J09-22, U07.1/2)	1490+ [368+] (6.0)	497 [18] (7.4)	1169 [55] (0.9)
Other respiratory (J23-99)	670 [98] (2.7)	526 [40] (7.9)	812 [100] (0.7)
Digestive (K)	751 [36] (3.0)	433 [11] (6.5)	2728 [26] (2.2)
Musculoskeletal (M)	408 [9] (1.7)	292 [7] (4.4)	1669 [14] (1.4)
Genitourinary (N)	573 [18] (2.3)	287 [<=5] (4.3)	1292 [28] (1.0)
External (S-Y, ex X60-84)	566 [<=5] (2.3)	301 [<=5] (4.5)	1476 [16] (1.2)

*Note: counts for cause specific deaths in square brackets are deaths which were not preceded by a previous hospitalisation in the same outcome category; these are the deaths that counted as outcome events in the cause-specific analysis, and not the total number of deaths from the given cause. *In the influenza group, only patients entering the study in 2019 were included in analysis of cause-specific outcomes, as linked cause of death data were only available from 2019 onwards. +In the COVID-19 group, 515/1122 (46%) hospitalisations in the COVID-19/Influenza/LRTI outcome category had COVID-19 codes (ICD-10 U07.1/2) and a further 461/1122 (41%) had pneumonia codes (J18) as the primary diagnosis; 342/368 deaths (93%) had COVID-19 as the underlying cause.*
