# Supplementary material for: Overall and cause-specific hospitalisation and death after COVID-19 hospitalisation in England: A cohort study using linked primary care, secondary care, and death registration data in the OpenSAFELY platform
Source: PLoS Med. 2022 Jan 25;19(1):e1003871. doi: 10.1371/journal.pmed.1003871 (PMC8789178; doi:10.1371/journal.pmed.1003871)
Supplement: S3 Table — (PDF) [file pmed.1003871.s009.pdf]

Accompanies *Bhaskaran et al. Overall and cause-specific hospitalisation and death after COVID-19 hospitalisation in England: a cohort study using linked primary care, secondary care and death registration data in the OpenSAFELY platform.*

S3 Table: Leading causes of death in COVID-19 and influenza groups

**COVID-19 group, N (%)** (2022 total deaths)		**Influenza group, N (%)** (558 total deaths*)	
COVID-19	500 (25)	Cancer	150 (27)
Cancer	359 (18)	Circulatory diseases	112 (20)
Circulatory diseases	339 (17)	Other respiratory (ICD 10 J23-99)	101 (18)
Other respiratory (ICD 10 J23-99)	156 (8)	Mental health/cognitive	35 (6)
Mental health/cognitive	150 (7)	Nervous system	32 (6)
		(Influenza)	≤5 (<1%)

**Only 558/1513 deaths in the influenza group are included in this table as the remainder (pre-2019) did not have cause of death data available (cause of death data were only available for 2019 onwards).*
