# Supplementary material for: Overall and cause-specific hospitalisation and death after COVID-19 hospitalisation in England: A cohort study using linked primary care, secondary care, and death registration data in the OpenSAFELY platform
Source: PLoS Med. 2022 Jan 25;19(1):e1003871. doi: 10.1371/journal.pmed.1003871 (PMC8789178; doi:10.1371/journal.pmed.1003871)
Supplement: Attachment — Submitted filename: plos med responses v1.1.docx [file pmed.1003871.s011.docx]

10^th^ November 2021

Dear Editors,

Thank you for the positive appraisal of our manuscript and the helpful further minor comments on our manuscript. We have made the changes requested, as outlined below. We are grateful to the editorial team and reviewers for helping us to strengthen our manuscript.

**Requests from Editors**

*Please trim the data access statement. If OpenSAFELY does not currently permit access consistent with PLOS' data policy (*[*https://journals.plos.org/plosmedicine/s/data-availability*](https://protect-eu.mimecast.com/s/iCnXCR1NGcgZzVGf0YRMg?domain=journals.plos.org)*) please state that and briefly explain the reason(s).*

**The statement has been trimmed as requested.**

*At line 47, please make that "February to December".*

**Done**

*At line 53, for example, please use the general style "13,733").*

**Abstract and Results numbers changed to this style.**

*At line 54, please make that "adjusted Hazard Ratio [aHR] 2.22 ..." and "aHR" can be used thereafter in the abstract.*

**Done.**

At line 55, should that be "aHR 0.95"?
**Yes thanks – corrected.**

At line 70, please make that "readmission or death" or similar.

**Done**

*In the abstract and throughout the text, please quote p values alongside 95% CI, where available.*
**P-values were added throughout the abstract and main results sections.**

*At line 307 and any other instances, please avoid "nearly 5-fold" in favour of quoting the actual value.*
**Changed as requested.**

*Please remove the information on data availability, funding and competing interests from the end of the main text. In the event of publication, this information will appear in the article metadata, via entries in the submission form.*
**These statements have been removed from the main manuscript file.**

*In the reference list, please correct the citations for references 10 and 11.*
**Sorry, these citations were removed at revision and the reference list did not update correctly. Now fixed.**

*Please ensure that journal names are abbreviated consistently (e.g., "Lancet" and derivatives rather than "The Lancet" etc).*
**Done**.

*We suggest breaking the analysis plan out into a separate attached file labelled "S1_Analysis_Plan" or similar, referred to in the Methods section (main text).*
**The study plan has been separated out from the rest of the appendix as suggested, and references in the text changed accordingly.**

Please adapt the label for the RECORD checklist to "S2_RECORD_Checklist" or similar, and refer to it by this name in the Methods section.

**Done.**

Comments from Reviewers:

*** Reviewer #1:

Thanks for the revised manuscript and replies to my original queries. The responses are comprehensive and apart from one small detail (in the flow diagram) I think this manuscript looks excellent and I recommend that it is accepted. I appreciate the work you have done in this revision - it makes the entire body of work robust.
The GP coverage information is helpful - I think acknowledging this as a limitation is all that's needed here. The additional details and tests about the proportional hazards are appropriate. The additional details and modified analysis (i.e. the ethnicity adjusted models) are appropriate and meet my initial query. The sensitivity analyses using Fine and Grey are a good addition and it's reassuring to see similar results to the Cox models.
**Thank you for the positive comments.**

Only one small check for Figure 1 - Is the number in the flowchart that died during hospitalisation or transfer (n=30,070) correct? This doesn't match the number before and after this exclusion was applied (108,308/75840).

**Thanks – this was an error and has now been corrected.**

*** Reviewer #3:

Thank you for addressing my comments.

My only remaining concern relates to the dataset coverage of "OpenSafely": in addition to mentioning data coverage as a limitation in the discussion section, the details of which 40% of the population are covered should be included in the Methods section as well.

**The methods section states that we used data from primary care practices using TPP SysmOne software. We have now further added a citation at this point to the paper (now reference 13) that describes the geographical distribution of GP software systems in England.**

We identified a few minor typographical errors in the "Why was this study done" section- line 86 "to compared", line 100 "by increased".

**Thanks, these typos have now been corrected.**

*** Reviewer #4:

The authors should be commended for addressing all of our concerns raised during review, including completing several additional analyses to test the robustness of their findings and adding an expanded discussion of the study limitations and policy applications. As a result, we believe that these steps served to substantially strengthen their conclusions which greatly improved the overall quality of the manuscript. Congratulations on completing this important work!

**Thank you for the positive comments.**

Yours faithfully,

Krishnan Bhaskaran
