# Supplementary material for: Overall and cause-specific hospitalisation and death after COVID-19 hospitalisation in England: A cohort study using linked primary care, secondary care, and death registration data in the OpenSAFELY platform
Source: PLoS Med. 2022 Jan 25;19(1):e1003871. doi: 10.1371/journal.pmed.1003871 (PMC8789178; doi:10.1371/journal.pmed.1003871)
Supplement: Attachment — Submitted filename: editor and reviewer responses at R3.docx [file pmed.1003871.s010.docx]

**Response to reviewer and editor comments**

We are grateful to all reviewers and the editorial team for the helpful feedback and suggestions, which we think have helped us to substantially improve the paper. Point by point responses to all comments are below.

**************

**EDITORS**

General comments:
E1. Generally, the style should be "We included 24,673 post-discharge COVID-19 patients ...", for example; numbers should be written out at the start of sentences

We have edited to the style above, in particular in the Abstract findings, and the opening of the Results section.

E2. Throughout the manuscript, please avoid inadvertent causal language e.g. “associated with higher risk” rather than “COVID-19 patients had higher risk...”

We agree with this sentiment, but would respectfully suggest that “COVID-19 patients had higher risk” is a descriptive statement of the numerical fact, and does not imply causality (in contrast, for example with “COVID-19 *increased* the risk…”. We have also checked other text with this in mind.

E3. Throughout the paper, please adapt reference call-outs to the following style: "... every year [1,2]." (noting the absence of spaces within the square brackets).

We have changed the style as requested.

E4. Data availability: PLOS Medicine requires that the de-identified data underlying the specific results in a published article be made available, without restrictions on access, in a public repository or as Supporting Information at the time of article publication, provided it is legal and ethical to do so. If the data are not freely available, please describe briefly the ethical, legal, or contractual restriction that prevents you from sharing it. Please also include contact information for data request enquiries (web or email address), noting that a study author cannot be the contact person for the data.

We describe in detail the information governance and ethics framework under which we accessed the data (“Information governance and ethics” section) and we have expanded our Data availability statement: “Detailed pseudonymised patient data is potentially re-identifiable and therefore not shared under our agreed information governance and ethics approvals (see above)”

E5. Title: Please revise your title according to PLOS Medicine's style. Please place the study design in the subtitle (ie, after a colon). We suggest “Overall and cause-specific hospitalisation and death after COVID-19 hospitalisation in England: a cohort study using linked primary care, secondary care and death registration data in the OpenSAFELY platform” or similar

We have accepted the suggested title.

E6. Abstract Methods and Findings: Please provide brief demographic details of the study population (e.g. sex, age, ethnicity, etc). Please include the study design in your Abstract. Please include the important dependent variables that are adjusted for in the analyses. Please specify that aHRs are presented with 95% CIs. Please provide numerical results to substantiate that “COVID-19 survivors with pre-existing dementia had higher risk of dementia death”. In the last sentence of the Abstract Methods and Findings section, please describe 2-3 of the main limitation(s) of the study's methodology.

We have addressed these requests.

**Changes**:

“…we conducted a cohort study, using linked primary care and hospital data in OpenSAFELY to…”

“We used Cox regression adjusted for age, sex, ethnicity, obesity, smoking status, deprivation, and comorbidities considered potential risk factors for severe COVID-19 outcomes.”

“COVID-19 patients had median age of 66 years, 13733 (56%) were male, and 19061 (77%) were of White ethnicity.”

“…in particular, COVID-19 survivors with pre-existing dementia had higher risk of dementia hospitalisation or death (age and sex adjusted-HR 2.47, 1.48-3.64)”

“Limitations of our study were that reasons for hospitalisation/death may have been misclassified in some cases due to inconsistent use of codes, and we did not have data to distinguish COVID-19 variants.”

E7: Abstract Conclusions: Please begin your Abstract Conclusions with "In this study, we observed ..." or similar, to summarize the main findings from your study without overstating your conclusions. Please address the study implications substantiated by the results.

Edited as requested.

E8: Author Summary. At this stage, we ask that you include a short, non-technical Author Summary of your research to make findings accessible to a wide audience that includes both scientists and non-scientists. The Author Summary should immediately follow the Abstract in your revised manuscript. This text is subject to editorial change and should be distinct from the scientific abstract. Please see our author guidelines for more information: [https://journals.plos.org/plosmedicine/s/revising-your-manuscript#loc-author-summary](https://protect-eu.mimecast.com/s/9_EcCWqVNfyg8BVhPUe1u?domain=journals.plos.org)
In the final bullet point of ‘What Do These Findings Mean?’, please describe the main limitations of the study in non-technical language.

An author summary has been added, as follows:

Why Was This Study Done?

-	Early studies have suggested that some people infected with SARS-CoV-2 may be at risk of developing health problems in the months after their initial infection. Given high rates of infection in many countries, this is a significant public health concern but there is currently limited evidence to inform policy.

-	The aim of this study was to systematically quantify the extent to which people who have been in hospital with COVID-19 may be at higher risk of dying or being readmitted to hospital, either overall or for specific illnesses, compared with people in the general population, and people who have been hospitalised with influenza.

What Did the Researchers Do and Find?

-	We used a cohort study design to compared risks of hospitalisation and death, overall and for a range of specific causes, between people who had hospitalised with COVID-19 (n=24,673), people with similar demographic characteristics in the 2019 general population (n=123,362), and people who had been hospitalised with influenza in 2017-2019 (n=16,058).

-	Compared with people in the general population, people who had had a COVID-19 hospitalisation were more than twice as likely to be re-hospitalised or die more than a week after discharge, with higher risks overall and for a range of specific causes,.

-	COVID-19 patients had broadly similar or lower risk of several outcomes compared with influenza patients, but risk of death overall, readmissions/death due to the initial infection, and dementia death were higher in COVID-19 patients.

What Do These Findings Mean?

-	Large numbers of people have been hospitalised with COVID-19 during the pandemic, and the raised risks of death and re-admission to hospital that we observed in these individuals could significantly impact public health and resources.

-	Risks might be minimised or mitigated by increase monitoring of patients in the months following hospital discharge, and greater awareness among patients and clinicians of potential problems.

E9. Introduction: Please indicate whether your study is novel and how you determined that, being careful to temper assertions of primacy.

We have added emphasis on novelty and that our study fills gaps in the evidence, in the final paragraph of the introduction; this immediately follows a passage in paragraph 2 on other papers on this topic and some of their key limitations, building the rationale for our study.

Change: “To generate new knowledge and fill gaps in the evidence base in this important emerging area, we therefore aimed to investigate the incidence of subsequent hospital admission and death, both overall and from a wide range of specific causes, following a COVID-19 hospitalisation in England.”

E10. Methods: Did your study have a prospective protocol or analysis plan? Please state this (either way) early in the Methods section. If a prospective analysis plan (from your funding proposal, IRB or other ethics committee submission, study protocol, or other planning document written before analyzing the data) was used in designing the study, please include the relevant prospectively written document with your revised manuscript as a Supporting Information file to be published alongside your study, and cite it in the Methods section. A legend for this file should be included at the end of your manuscript. If no such document exists, please make sure that the Methods section transparently describes when analyses were planned, and if/when reported analyses differed from those that were planned. Changes in the analysis-- including those made in response to peer review comments-- should be identified as such in the Methods section of the paper, with rationale. If a reported analysis was performed based on an interesting but unanticipated pattern in the data, please be clear that the analysis was data-driven.

No formal protocol was developed for this study though a brief outline study plan was created in February 2021 setting out the overall objectives and approach (now added as an appendix). This approach evolved during discussions about the study, with all major design decisions being taken prior to analysing the data, except the decision to exclude the first week of follow-up after hospital discharge, which was done when it became clear from initial descriptive analyses that relatively large numbers of rapid re-hospitalisations/deaths observed during this time were likely to obscure the longer term outcomes that were of most interest. We have now noted this in the methods.

**Change**s:

“A brief outline protocol was developed to set out the aim and approach (presented in supplementary file S1 (appendix), along with an outline of changes and justification).” (Methods, study design and study population)

“A brief outline study plan was created in February 2021 setting out *a priori* the aim and overall approach for the present study; the design was developed further in discussion with the study team prior to data analysis (supplementary file S1 (appendix)).” (Methods, study design and study population)

We selected ..individuals … who were alive and under follow-up in a TPP practice one week after discharge (to avoid a focus on hospital transfers and immediate readmissions/deaths, as early descriptive data suggested a large number of outcomes in week 1 would have obscured the longer-term outcomes that were of primary interest).” (Methods, study design and study population)

Original outline plan and summary of changes and justification added to supplementary file S1 (appendix).

E11. Please ensure that the study is reported according to the RECORD guideline, and include the completed RECORD checklist as Supporting Information. Please add the following statement, or similar, to the Methods: "This study is reported as per the REporting of studies Conducted using Observational Routinely-collected Data (RECORD) guideline (S1 Checklist)." The RECORD guideline can be found here: [https://www.record-statement.org/](https://protect-eu.mimecast.com/s/a-tyCX5WOIMyZYWc8fHqS?domain=record-statement.org) When completing the checklist, please use section and paragraph numbers, rather than page numbers which will likely no longer correspond to the appropriate sections after copy-editing.

RECORD checklist completed

E12. Results: Please incorporate Figure A1 into the main paper. Where adjusted analyses are presented, please also provide the unadjusted analyses, and indicate which factors are adjusted for (e.g. please list comorbidities) in the tables/figure legends.

Done

E13. Discussion: Please present and organize the Discussion as follows: a short, clear summary of the article's findings; what the study adds to existing research and where and why the results may differ from previous research; strengths and limitations of the study; implications and next steps for research, clinical practice, and/or public policy; one-paragraph conclusion.
Please remove all subheadings within your Discussion e.g. Key findings

Done and subheadings deleted.

E14. Tables: Please define abbreviations used in the table legend of each table e.g. GFR (Table 1)
Table A1 - please specify what is represented by numbers in brackets ( )

We have added a definition for GFR in the table footnotes. We have edited in Table A2 (new numbering) to clarify that the numbers in brackets are %s.

E15. References: Please ensure that journal name abbreviations match those found in the National Center for Biotechnology Information (NCBI) databases, and are appropriately formatted and capitalised. Please also see [https://journals.plos.org/plosmedicine/s/submission-guidelines#loc-references](https://protect-eu.mimecast.com/s/Os26CY58PI6JXxwFWTmA4?domain=journals.plos.org) for further details on reference formatting.

Checked.

E16. Supplementary files: Please see [https://journals.plos.org/plosmedicine/s/supporting-information](https://protect-eu.mimecast.com/s/zQQ3CZ01QioVYN0IZrCzp?domain=journals.plos.org) for our supporting information guidelines.

Checked.


Comments from the reviewers:

Reviewer #1:

Thanks for the opportunity to review your manuscript. My role is a statistical reviewer so my comments focus on the design, analysis, and data in this manuscript. I've put overall comments first followed by queries relevant to a specific section of the manuscript (with a line reference).

1.1 This is an interesting and important study. Routinely collected health data was used to create a cohort that enable a comparison of medium-term outcomes in patients hospitalised for COVID-19, to hospitalised influenza patients and general population (patients registered in the GP system). The COVID patients were directly matched to the comparators based on age (within 3 years), sex, area, and calendar month. Three outcomes were examined - all-cause mortality/first hospitalisations, all-cause mortality, and cause-specific hospitalisation. COVID patients had higher risk of the composite outcome than general population, but similar rates to the influenza in-patients. COVID patients had higher mortality than the other groups, and similar risk of cause-specific apart from subsequent infection or other respiratory infection and mental health/cognitive related admissions/deaths.

The manuscript is well-written and clearly described (particularly given the complexities of assembling and analysing the data). As a side-note the fact that it is possible to do this study so quickly shows the value of setting up the capacity for linked health data to be used in research and is very impressive (I am jealous you have such timely access to this data).

Thank you for the positive comments and we agree that timeliness was an important advantage of the platform and data source available for this study.

1.2 The GP records were used to create the 'general population group' - is it effectively universal for everyone to be on this database, is there the possibility that this could miss patients who can't/don't seek healthcare?

Around 98% of the population are estimated to be registered with a GP in England, so the proportion missed due to not seeking healthcare is likely to be minimal. This point has been added to the discussion. The particular database used in this study covers one particular GP software provider, covering about 40% of the population, as mentioned in the Discussion.

**Change**: “Ninety-eight percent of the population are registered with a general practice,^26^ minimising selection biases due to health-seeking behaviours, and our data source covered around 40% of the population of England, giving us high statistical power…” (Discussion, strengths and limitations)

1.3 L106. The use of the general population comparator is clear, perhaps worth clarifying here in the aims that the influenza hosp. population addresses the issue of potential for increased risk of events after any hospital exposure.

Thank you for the suggestion; we have added this point about the reason for including an influenza comparator.

**Change**: “We aimed to compare post-COVID risks with two separate comparison groups: (i) the general population, and (ii) people hospitalised for influenza prior to the current pandemic. The latter was included to provide a comparison with risks after hospitalisation in general, using admissions from a well-characterised infectious disease.” (Introduction)

1.4 L114. Is the 40% sample broadly representative? Do some regions contribute more than expected than from their relative population levels?

The 40% sample is somewhat geographically clustered, due to distribution of different GP software systems in different regions. We have added this point to the discussion.

**Change**: “…our data source covered around 40% of the population of England, giving us high statistical power, though it should be noted that our study population would not have been geographically representative of England, since TPP SystmOne software is more widely used than other systems in parts of Eastern and Southern England, and used less than other software in London.^27^” (Discussion, strengths and limitations)

1.5 L128. What was the rationale of matching to the same calendar date a year earlier rather than the same year? Was this because of availability of linked mortality data? This does make the assumption that overall mortality and hospitalisations rates of those not affected by COVID or influenza were similar in 2019 to 2020 - is there any information you can provide that supports this?

We matched to the same calendar date a year earlier because we wanted to obtain a comparison of outcomes with those in “normal” pre-pandemic times. We decided not to include a comparison with general population data from the same calendar year because even in the general population without a COVID-19 hospitalisation, any adverse outcomes could be directly related to (non-hospitalised) COVID-19 infection, or indirect pandemic-related factors (e.g. reduced care for non-COVID conditions), complicating interpretation. We have now clarified this rationale in the paper.

**Change**: “Two comparison groups were also selected. First, we identified people under follow-up in the general population in 2019, individually matched 5:1 to the COVID-19 group … The rationale for matching to 2019 data was to provide a comparison with routinely faced risks during pre-pandemic times.” (Methods, study design and study population)

1.6 L129. Are the ICD-10 codes used here the same as presented in Table A1?

To clarify, yes the ICD-10 codes listed denoting outcomes in paragraph 1 of “Methods/Outcomes and Covariates” are the same as those in column 1 of Appendix Table A2 (new table numbering), which gives the distribution of the same outcomes across the comparison groups.

1.7 L134. Were the hospitalisations used to derive the outcomes 'acute', e.g. would this outcome include in-patient episodes for planned procedures like cataract surgery or just unplanned admissions?

There was no distinction between planned and unplanned admissions in our analysis, or in our source data, and our outcomes would have included all admissions relevant to each outcome. We added a clarification on this.

**Change**: “Hospitalisations were identified from linked SUS data and included all admissions (whether planned or unplanned).” (Methods, outcomes and covariates)

1.8 L164. Why was the 8th day after discharge used for COVID and Influenza patients? Also, do influenza patients have similar in-hospital stays to the COVID patients? E.g. time spent in ICU, time on mech ventilation, overall LOS?

Follow-up started on the 8^th^ day after discharge because we were wished to avoid the analysis being dominated by week 1 readmissions/deaths which might represent continuation of the initial acute disease despite the decision to discharge, or which might actually reflect hospital transfers (which can appear in the data as discharge followed by rapid readmission). Large numbers of such outcomes were apparent in early descriptive analyses. We have now expanded on this in the methods.

Table 1 presents length of hospital stay and proportion with critical care in the COVID-19 and influenza-hospitalised groups. COVID-19 hospital stays were longer and more likely to involve critical care than influenza hospitalisations. This is mentioned in the discussion (“descriptive data showed that COVID-19 patients tended to have longer hospital stays and more critical care than those hospitalised for influenza.” [Discussion, strengths and limitations]) We did not have data on time spent in ICU or time on mechanical ventilation.

**Change**: “We selected ..individuals … who were alive and under follow-up in a TPP practice one week after discharge (to avoid a focus on hospital transfers and immediate readmissions/deaths, as early descriptive data suggested a large number of outcomes in week 1 would have obscured the longer-term outcomes that were of primary interest).” (Methods, study design and study population)

1.9 L174. The K-M graphs seem to show proportional hazards for comparisons between the key groups, how was this assumption checked during the analysis for these, and also the other covariates (and the form of any continuous covariates in the analysis)?

We agree that proportional hazards deserved more consideration and we also took on board the comment from reviewer 4 (comment 4.1) that increased risks after hospitalisation may be more pronounced early on. We investigated this by fitting interactions between exposure and follow-up time, and detected that for some outcomes a more strongly raised risk was indeed seen earlier in follow-up. We have now reported this important extra insight in the paper.

We have also added extra checks with regards to non-proportionality of adjustment covariates. We checked for a zero slope in the Schoenfeld residuals for each adjusted model, and where evidence of non-proportionality was found, we conducted a sensitivity analysis adding an interaction term between follow-up time and each variable for which evidence of non-proportionality was found; this had little effect on hazard ratios and results are presented as part of a new figure showing sensitivity analyses in the appendix.

**Changes**: “Interactions with follow-up time (classified as <30 days, 30-<90 days and ≥90 days from hospitalisation [COVID-19/influenza groups] or entry [general population controls]) were examined to investigate whether any increased risk was concentrated in early follow-up, and as an implicit check of proportional hazards. We also checked for proportional hazards in adjustment covariates by testing for a zero slope in the Schoenfeld residuals for each adjusted model; where there was evidence of non-proportionality, an interaction between follow-up time and any variables with evidence of non-proportional hazards was added to the model as a sensitivity analysis.” (Methods, Statistical analysis)

“We found evidence of changes over time in the hazard ratios of several outcomes, with more pronounced raised risks earlier following COVID-19 hospitalisation (Appendix Figure A2). Our results changed little in a range of sensitivity analyses including …adjusting for non-proportional hazards in adjustment variables … (Appendix Figure A3)” (Results)

“Risks were higher for all categories of disease-specific hospital admissions/deaths after a COVID-19 hospitalisation than in general population controls, with excess risks more pronounced earlier in follow-up for several outcomes.” (Discussion, key findings)

Figure A2 added to show the results of models containing interaction between exposure and follow-up time.

Figure A3 added which includes results of sensitivity analyses adding time interactions to account for non-proportional hazards in covariates.

1.10 L177. How was age included in the model, as a categorical variable or continuous?

Age was included as continuous; we used a 4-knot cubic spline to capture any non-linearity, but for the lower-powered cause-specific outcome analyses we simplified this to a linear term to improve estimation. We have now noted this in the text.

**Change**: “Separate models were fitted for the comparisons with matched 2019 general population controls (models stratified by matched set), and with influenza controls (models adjusted for age [continuous, as a 4-knot restricted cubic spline except in cause-specific outcome models where a simpler linear term was used due to lower power], sex, STP and calendar month).”

1.11 L180. This is certainly true, but there hasn't been any information presented to demonstrate that MAR is a likely mechanism of missingness. The number excluded from each of these models should be presented, and with a comparison of characteristics by missingness. This does also make the interpretation of the difference between the adjusted and non-adjusted models more difficult as they are being done on different samples. In the previous publication using OpenSAFELY, an imputation model was used to handle the missing ethnicity - could this be applied for these analyses as well?

Thank you for these important comments. We agree that multiple imputation could be reasonably applied to these data to handle the missing ethnicity, as in previous OpenSAFELY work. We have now rerun all ethnicity-adjusted models using this approach. A population-calibrated imputation that we carried out in a previous publication (Williamson et al, Nature 2020) suggested minimal non-random missingness (calibration parameters close to zero) suggesting that MAR is a reasonable assumption for this variable. We do not think missing BMI and smoking are likely to be MAR because smokers or people outside the healthy weight range are more likely to have their smoking status/weight recorded in primary care, so we stuck to a complete case approach for these data. The complete case assumption is untestable because one would need to condition on the missing values themselves, but we saw no reason that recording of BMI/smoking in primary care would have been independently associated with the study outcomes. In fact, there were relatively few missing data for these variables (missingness <2% in each group for smoking, and <8% for BMI) so that any violation of the assumption would have had relatively little impact. We have added some demographic data comparing those with and without missing smoking/BMI data to the appendix. We also agree that comparison across adjusted/unadjusted models is difficult when the study population is changing; we have therefore excluded those with missing smoking/BMI from all regression models (as presented in Figures 3 and 5), regardless of the adjustments in individual models. This means that all HRs are estimated on the same patients. The numbers excluded from regression models have been added to the Figure 3/5 footnotes.

**Changes**:

“Missing ethnicity was handled using multiple imputation (10 imputations) based on a multinomial logistic model including all covariates from the substantive models and an indicator for the outcome of interest; a population-calibrated multiple imputation carried out in a previous analysis in this data sources showed minimal non-random missingness in ethnicity data (calibration parameters were close to zero) suggesting missing at random to be a reasonable assumption.^6^ People with missing data on ~~ethnicity~~ body mass index or smoking were excluded from regression models ~~that used these variables~~. Such a “complete case analysis” is valid under the assumption that missingness is conditionally independent of the outcome;^18^ whilst this assumption cannot be verified in the data (because one cannot condition on the missing values themselves), we had no reason to doubt that recording of BMI/smoking in primary care would have been independently associated with the study outcomes; on the other hand we deemed the missing at random assumption required for multiple imputation to be unlikely to hold for these variables in primary care (e.g. because smokers or those at the extremes of the weight distribution are more likely to have these data recorded).” (Methods, Statistical analysis)

“Body mass index and smoking were 93-99% complete in all groups; those with missing data on these variables (who were excluded from later regression modelling) were more likely to be younger, male, and from more deprived areas (Appendix Table A1). Missing ethnicity (which was handled by multiple imputation) was <2% in the COVID-19 and influenza groups, but 25% in the matched control group (no hospital-based ethnicity records were available for this group).” (Results)

Footnotes added to figures 3/5 (revised figure numbering) to denote number excluded due to missing BMI/smoking data

New Appendix Table A1 added, showing demographic characteristics of those excluded from regression analyses due to missing BMI/smoking data.

1.12 L185. What software was used for these analyses and data management?

We have added this information to the methods.

**Changes**: “Data management and analysis was carried out in Python version 3.8 and Stata version 16.” (Methods, last paragraph))

1.13 L185. Censoring for death assumes that the cause-specific event would be independent of mortality, which seems unlikely. Typically for this type of analysis I would use a Fine-Grey model rather than K-M, although the interpretation of effect estimates from F-G is little more complicated than K-M. Would you consider that censoring is independent from the hospitalisations here, and did you do any sensitivity analyses to check if the K-M estimator is reasonably here?

We only used the Kaplan-Meier estimator for the composite (overall hospitalisation or death) and overall mortality outcomes, where there was effectively no competing risk. Cumulative incidences for cause-specific outcomes (i.e. Figure 4) were estimated using an appropriate estimator that does not censor the competing risks. We have added a reference to this paper.

For regression modelling, we agree that the Fine and Gray model would have been an alternative choice, with a different (and as noted, slightly more complex) interpretation. We felt that analysis of the cause-specific hazards was of primary interest, essentially characterising the risk among those still “at risk” at any given moment (Austin et al, Circulation 2016, doi 10.1161/CIRCULATIONAHA.115.017719). We agree that there is an implicit assumption of independent censoring which is untestable. Only deaths from a different category of cause to the outcome of interest are censored in this approach, making the assumption of independence more reasonable, but it is still possible that there was some association between censoring and outcome risk. The overall proportion of the study population censored for competing deaths was relatively small (ranging from 1.4 to 2.6% for different cause-specific analyses), so our analyses should have been relatively robust to any such non-independent censoring. Fine and Gray subdistribution hazard ratios avoid the independent censoring assumption, but have a different interpretation, characterising differences in the cumulative incidence of specific outcomes, even where mediated through changes in the risk of competing outcomes, and of less relevance to characterising immediate risk among “survivors”. As both analyses are informative in their own right and have different strengths and limitations in terms of assumptions and interpretation, we decided to repeat the modelling using Fine and Gray regression and include this as a secondary analysis. The results are presented in the appendix and show similar patterns to the Cox analysis.

**Changes**: “Cumulative incidence of cause-specific hospitalisation/death outcomes were calculated with deaths from other causes treated as a competing risk.^19^” <reference added> (Methods, statistical analysis)

“In a secondary analysis, we fitted Fine and Gray regression models to characterise overall differences in the cumulative incidence of cause-specific outcomes in the presence of competing risks.” (Methods, statistical analysis)

“In secondary analyses using Fine and Gray models, subdistribution hazard ratios were similar to cause specific hazard ratios from the primary Cox models (Appendix Figure A3).” (Results)

“Our analysis of cause-specific outcomes made an assumption of independent censoring, but deaths from competing outcomes were censored and may have been related to risk of the outcomes under study; our results are likely to have been robust to some violation of independence because the proportion of patients censored due to death from other causes was low (ranging from 1.4-2.6% of the study population for specific analyses). Fine and Gray modelling (which does not censor competing events) showed a similar pattern of results to the primary analysis.” (Discussion, strengths and limitations)

Figure A3 added which includes results from Fine and Gray models for cause-specific outcomes.

1.14 L263. Using influenza is clever but it does assume that the hospital stay for influenza and COVID patients is similar - I think this is something that should be acknowledged or some more context provided so it's clear how comparable these patients are.

We did not intend any assumption that hospital stays for COVID and influenza patients were similar, or that patients were equally sick. In fact we included in Table 1 data on length of hospital stay and use of critical care for the two hospitalised groups, showing that the COVID-19 group had longer hospitalisations and more critical care use than the influenza group, as noted in the results (“COVID-19 patients had longer median duration of hospital stay and were more likely to have received critical care during their admission than influenza patients.” [Results]) This reflects the now well-established higher risk of serious disease with SARS-Cov-2 compared with influenza infection. We still think the influenza comparison group is useful. First, as noted by the reviewer above (comment 1.3), this comparison group helps to account for the general raised risks associated with any hospital admission. Secondly, influenza is a related infectious disease and has been used as a benchmark for measuring COVID-19 risks and impacts since the start of the pandemic. We further expanded on the rationale for this comparison group in the introduction (as requested in comment 1.3).

**Change**: “We aimed to compare post-COVID risks with two separate comparison groups: (i) the general population, and (ii) people hospitalised for influenza prior to the current pandemic. The latter was included to provide a comparison with risks after hospitalisation in general, using admissions from a well-characterised infectious disease.” (Introduction)

1.15 Figure A1. There is small cell suppression on the consort flow-chart (second box of exclusions) - was this a condition of the accessing the data? It's not something I'm used to, but I can still make sense of what is happening and reconcile the numbers so I think this is ok.

Small number suppression (for cell counts ≤5) has been implemented in all OpenSAFELY outputs as an information governance safeguard to reduce the risk of reidentification.

1.16 Figure 1. These figures are ok for review purposes but they could be smartened up a bit, e.g. directly labelling the cum incidence curves instead of the legend would make it easier to read (the legend takes up ~25% of the plot area), maybe colour in addition to line type, label on the Y axis

We have added the y-axis label. We prefer the separate legend for clarity and have avoided colour due to the potential for greyscale printing, however we are happy to discuss these details further with the production team.

1.17 Figure 2. Again - perfectly readable, but maybe a few tweaks (e.g. x axis goes out to 20 when there doesn't seem to be any CIs that reach that level), also the null effect line overwrites some of the labels.

Thank you for the suggestions. The x-axis was revised to go out to 10 only, and the null effect line was shortened to avoid overlap with the headings.

Reviewer #2: Rosie Cornish
This study provides important evidence about outcomes following hospitalisation due to COVID-19. I only have a few comments.

2.1. The authors have used a complete case analysis, which is justified as being appropriate provided missingness is conditionally independent of the outcome. Did the authors investigate this? It seems like a plausible assumption, but it would be helpful to state somewhere whether the data supported this.

Please see also our detailed response on missing data at Reviewer 1 Comment 1.11. We now employ multiple imputation to deal with missing ethnicity as suggested by Reviewer 1 (we believe missing at random [MAR] to be a reasonable assumption for this variable). We still use complete case analysis for missing BMI and smoking data because MAR is unlikely for these variables. The assumption that complete case analysis makes about conditional independence is untestable by definition, because one would have to condition on the missing values themselves, but we have expanded on why we believe the assumption to be reasonable. The analysis is likely to be quite robust to violation of this assumption due to the relatively low level of missingness in these variables (<2% for smoking, <8% for BMI).

**Changes**:

“Missing ethnicity was handled using multiple imputation (10 imputations) based on a multinomial logistic model including all covariates from the substantive models and an indicator for the outcome of interest; a population-calibrated multiple imputation carried out in a previous analysis in this data sources showed minimal non-random missingness in ethnicity data (calibration parameters were close to zero) suggesting missing at random to be a reasonable assumption.^6^ People with missing data on ~~ethnicity~~ body mass index or smoking were excluded from regression models ~~that used these variables~~. Such a “complete case analysis” is valid under the assumption that missingness is conditionally independent of the outcome;^18^ whilst this assumption cannot be verified in the data (because one cannot condition on the missing values themselves), we had no reason to doubt that recording of BMI/smoking in primary care would have been independently associated with the study outcomes; on the other hand we deemed the missing at random assumption required for multiple imputation to be unlikely to hold for these variables in primary care (e.g. because smokers or those at the extremes of the weight distribution are more likely to have these data recorded).” (Methods, Statistical analysis)

2.2. Care home status is listed in Table 1 but it is not mentioned as a variable included in the fully adjusted models. Since this is a factor strongly associated with COVID-19 as well as the outcomes, I think it should be included as a covariate.

Our classification of care home residence has been shown to have good positive predictive variable, but limited sensitivity, therefore we think there is a relatively high risk of misclassification in this variable. For this reason, we preferred to omit the variable from our main modelling, but we have added adjustment in a new sensitivity analysis and the results were very similar to the primary analysis. We have added this information to the paper.

**Changes**:

“We also extracted information on residence in a care home as described previously; this was used in descriptive and sensitivity analyses only due to limited sensitivity.^18^” (Methods, outcomes and covariates)

“Further sensitivity analyses included … adjusting for care home residence.” (Methods, statistical analysis)

“Our results changed little in a range of sensitivity analyses, including … adjusting for … care home residence (Appendix Figure A3).” (Results)

“Our findings were robust in a range of sensitivity analyses.” (Discussion, strengths and limitations)

Figure A3 added which includes results of sensitivity analyses adding adjustment for care home residence.

2.3. In the methods the authors state that COVID-19 discharges were matched on age, sex, geography and time (month). It might therefore be helpful to explain in the text why the distribution of entry dates for COVID-19 discharges does not match the distribution among the general population controls as shown in Figure A2.

Thank you for spotting this. It is correct that since almost all COVID-19 discharges were successfully matched, including on calendar month, the distributions over time of the COVID-19 and general population groups should have been similar (but displaced by one year). On further investigation we confirmed that the distributions were in fact very similar as expected, but the default bins in the histogram were misleadingly exaggerating small differences that arose due to our matching on calendar month rather than exact calendar date (this was done because of limitations of the platform). We have now corrected the histogram bins; the matches now fall as expected within the equivalent monthly bin as the corresponding COVID-19 patient, showing that the seasonal distributions of entry dates in the two groups do indeed reflect the matching.

**Change**: Appendix Figure 1 corrected.

2.4. It would be helpful to indicate on Figures 2 and 4 the numbers included in each analysis so readers can see how the sample sizes for the different models differ.

We have added footnotes to figures 3/5 (revised figure numbering) to denote number included/dropped due to missing BMI/smoking data. Exclusions are now consistent across models with different levels of adjustment, as suggested by reviewer 1 (comment 1.11).

**Changes**: Footnotes added to Figures 3 and 5 showing numbers included/excluded in models.

2.5. In the results section the authors state that people in the COVID-19 group had similar or lower risk of admission or death from most causes, whereas in the abstract they say that most risks were similar (i.e. "or lower" is omitted). I think it is important to state this in the abstract as well as in the results.

We agree and have made the suggested change.

**Change**: “Risks for cause-specific outcomes were higher in COVID-19 survivors than general population controls, and largely similar or lower in COVID-19 compared with influenza patients.” (Abstract results)

2.6. Figure 1 states that there were 79,502 matched controls but a figure of 123,362 is given elsewhere.

Thanks for pointing out the discrepancy. This was a version control error with the Figure, and the correct number is 123,362, as per Table 1 and elsewhere. We have now corrected this.

**Changes**: Figure 1 flow chart corrected.

2.7. The final point on Figure 1 states "(6,689 were discharged in 2019 and had linked data registration available)". I think this should read "… and had linked death registration data available".

Thank you for pointing this out, we have corrected the error.

**Change**: typo in Figure 1 corrected.

Reviewer #3:

3.1 This study used routinely collected health administrative data covering 40% of England's population to examine medium to long-term hospitalization and mortality following acute COVID-19. They included 24,673 patients discharged between February and December 2020 who survived 8 days without readmission. Comparison groups were: (i) matched patients in the general population in 2019, and (ii) patients discharged from hospital with influenza (2017-2019). Although incidence of subsequent hospitalisation or death in the COVID-19 group was higher than in general population controls, incidence was comparable to the influenza group (34.8%, 15.2%, and 37.8%, respectively). Compared to the influenza group, the COVID-19 group had higher all-cause 6-month mortality (7.5% vs. 4.9%), and greater risk of death for patients with dementia (HR 2.32). This study demonstrates the importance of selecting appropriate control groups and presents a new finding on the association between COVID-19 and dementia.

This paper was well-written and quite succinct. The topic of long-term outcomes after COVID-19 is highly topical and important given how many people have had COVID-19 infection. The inclusion of a hospitalized influenza control group (from before the pandemic) was appropriate, and consistent with other research (Verma AA et al. CMAJ March 22, 2021 193 (12) E410-E418).The stratified analyses around the dementia outcome is appreciated- this is helpful to address possible confounding related to care home residence.

Thank you for the positive comments.

Major comments:

3.2 The authors should further elaborate on the relevance of the primary research question - the introduction and discussion should make a case for why this study needed to be done, beyond just "to strengthen the evidence base" (line 102). Furthermore, the discussion needs further detail on how the results might be useful, and what further research would be most valuable here.

Thank you for the suggestion. To the extent possible within the constraints of space, we have now elaborated further on the rationale and importance of the study, and the need for further research. Reviewer 4 (opening comments) notes that the study has “clear applications to healthcare resource planning and policy in the care of these individuals suggesting a substantial extra burden on healthcare systems in the future”, and we have tried to make this clearer.

**Changes**:

“Given high rates of current and past SARS-CoV-2 infection in many countries, understanding risks to health beyond acute infection is vital to support resource planning and inform measures to mitigate and reduce risks. To generate new knowledge and fill gaps in the evidence base in this important emerging area, we therefore aimed…” (Introduction).

“These findings suggest a need for services to support and closely monitor people following discharge from hospital with COVID-19, for example through more frequent/active follow-up in primary care in the weeks and months following a hospitalisation. Our results can be used to help inform healthcare providers and raise awareness of potential complications during this period. Our findings will also help with public health resource planning in the context of high rates of SARS-CoV-2 infection in many countries. Ongoing monitoring will be important to investigate whether these patterns persist in the light of new variants and increasing levels of vaccination.” (Discussion, conclusion)

3.3 Further discussion is also warranted about why COVID patients had more COVID readmissions in the context of published data on long COVID symptoms as well as persistence of viremia in some cases. These are possibly important differences with influenza that explain the findings.

We have moved some of the discussion around early evidence on long COVID to the discussion (per comment 3.5). We have also mentioned the possibility of persistent viraemia being linked to the high rates of COVID-19 deaths in the COVID-19 group.

**Changes**:

“Our findings are consistent with emerging evidence from early studies suggesting that a subset of people infected with SARS-CoV-2 can experience health problems for at least several months after the acute phase of their infection, with fatigue, pain, respiratory and cardiovascular symptoms, and mental health and cognitive disturbances being among the problems that have been frequently described under the term “post-acute COVID-19 syndrome” [10]; however, epidemiological characterisation of such sequelae has been limited. Small descriptive studies of COVID-19 survivors have been suggestive of high incidence of a range of outcomes including respiratory, cardiovascular, and mental-health related [11,12]; the present study puts helps to contextualise these observations by adding explicit comparison with risks experienced by the general population, and by people with a recent influenza hospitalisation.” (Discussion, findings in context; n.b. edited version of a passage that was moved from the Introduction)

“A quarter of deaths after a COVID-19 hospitalisation had COVID-19 listed as the underlying cause, but it is not clear from our data whether patients experienced specific complications after hospital discharge that were then attributed to COVID-19, and the possibility of persistent viraemia in these patients cannot be excluded from our data.” (Disussion, findings in context)

3.4 The findings with respect to dementia are interesting however what is missing is discussion of the relationship between COVID, delirium and dementia. We know that delirium episodes often worsen dementia, and that it is also often difficult to distinguish clinically between subacute/chronic delirium and progressive dementia. Hence it is quite possible that the signal related to dementia outcomes reflects COVID-related delirium (which is well described), either directly or indirectly. The other important consideration is that those who were diagnosed with COVID were isolated (presumably to a greater extent than those with influenza), and a lack of interpersonal contact is also known to worsen dementia/delirium outcomes.

Thank you for these thoughtful points. We have added the point on delirium to the passage discussing the dementia findings. We mentioned a potential role for social isolation (“…acute COVID-19 and associated hospital admission, social isolation, and medications may have accelerated progression of patients’ dementia…” – Discussion, findings in context)

**Change**: “COVID-19-related delirium may have also triggered or worsened emerging dementia in some patients, or even driven a degree of misclassification given the potential clinical challenge in distinguishing between subacute or chronic delirium and progressive dementia.” (Discussion, findings in context)

Other suggestions for improvement:
Introduction
3.5 the middle paragraph has a lot of information about other literature which would be better placed in the discussion- I suggest reducing the amount of detail in here and instead making a stronger case for why this research needed to be done.

We have moved some of the text from the middle paragraph of the introduction into the discussion (with some minor editing), as suggested. (Please see changes in response to comment 3.3 for details)

Methods
3.6 The authors point to a previous paper describing the OpenSAFELY program, but I think some more details on this are needed. For example, which 40% of the population does it cover? Is this percent of the population generalizable to the other 60% of the population? I ask this because the overall sample appears low (108,308) compared to the overall number of people who have been diagnosed with COVID-19 in England

Our data cover primary care practices that use one particular electronic health record software system (TPP SystmOne), which is used by approximately 40% of GPs. The sample is somewhat geographically clustered, due to popularity of different GP software systems in different regions. We have added this point to the discussion. With regard to the number of cases included here, please note that we only included hospitalised cases, and not all those diagnosed with COVID-19. As a check, we looked at hospitalisation statistics for the UK published online (<https://ourworldindata.org/covid-hospitalizations#what-is-the-weekly-number-of-new-hospitalizations-due-to-covid-19>) which suggest around 270,000 new hospitalisations for COVID-19 in 2020, so the figure of 108,308 is roughly as expected for 40% of England.

**Change**: “…our data source covered around 40% of the population of England, giving us high statistical power, though it should be noted that our study population would not have been geographically representative of England, since TPP SystmOne software is more widely used than other systems in parts of Eastern and Southern England and , and used less than other software in London.^27^” (Discussion, strengths and limitations)

3.7 what does "under follow-up" mean? (ln. 120)- does this just mean they had not yet experienced an outcome? Please clarify.

Sorry this was not clear. We meant alive and registered with a GP using TPP software (and thus represented in our data source). We have added a clarification.

**Change**: “We selected … individuals … who were alive and under follow-up in a TPP practice…” (Methods, study population)

Results

3.8 what was the median, and minimum observation time for outcomes across patients? I see that the maximum was 315 days. This question also relates to what "under follow-up" means- how complete was the follow-up? Since this sounds like health administrative data, my expectation was that one would only be lost to follow-up if they moved away, but this should be clarified.

COVID-19 patients were considered “under follow-up” in the analysis from the 8^th^ day after hospital discharge until occurrence of the outcome under study, or the administrative censoring date for the relevant source of outcome data (i.e. the date of latest available data). In addition, for all-cause mortality (ascertained from primary care data), follow-up ended if patient moved outside the network of practices using TPP software. This is outlined in more detail in Methods, statistical analysis (“Follow-up began on the 8^th^ day after hospital discharge for the COVID-19 and influenza groups, and on the 1^st^ of the same calendar month in 2019, for the general population control group. Follow-up ended at the first occurrence of the analysis-specific outcome, or the earliest relevant censoring date for data availability/coverage for the outcome being analysed… For outcomes involving hospital admissions the administrative censoring date (for SUS data) was 30^th^ December 2020; for outcomes involving cause of death, the administrative censoring date (for ONS mortality data) was 11^th^ March 2021; for the all-cause death outcome which was ascertained in primary care data, patients were censored at date of deregistration if they had left the TPP general practice network.”)

The median follow-up time in the COVID-19 group (i.e. time considered at-risk for the outcome) was therefore different for each outcome. For the composite outcome (time to first hospitalisation or death) median follow-up was shortest at 61 days (because any hospitalisation or death ended “time at risk” in this analysis), while for all-cause mortality, median follow-up was longer at 167 days because follow-up was not terminated by hospitalisation. For cause-specific outcomes, median follow-up ranged from 91 to 108 days. We have clarified this information to the paper. Please note that since there were two main pandemic “waves” in England in 2020 (Appendix Figure A1), these figures are a little misleading in isolation as they mask a bimodal distribution for follow-up, with a significant proportion of patients followed-up for considerably longer than the median. For example, though median follow-up for death overall was 167 days, there were two peaks, with wave 1 patients having follow-up distributed around ~300 days and wave 2 patients having follow-up distributed around ~100 days; 44% of patients (largely from “wave 1”) had at least 250 days of follow-up for this outcome.

Patients having an outcome on the day of entry were assumed to have 0.5 days of follow-up, which was the minimum possible follow-up in the study for all outcomes.

**Changes**:

“Median time at risk in the COVID-19 group was 61 days for the composite outcome, and 167 days for death; total time at risk followed a bimodal distribution corresponding to the two main pandemic waves in England.” (Figure 3 footnote)

“Median time-at risk in the COVID-19 group ranged from 91 to 108 days across outcomes; total time at risk followed a bimodal distribution corresponding to the two main pandemic waves in England.” (Figure 5 footnote)

3.9 tables and figures were very helpful and nicely done

Thank you.

3.10 I note that the COVID group experienced fewer cancer-related outcomes than the influenza group, however this was not mentioned in the results text. I think this finding is worthy of some discussion (for discussion section as well)- could this be because the COVID group had lower rates of cancer at baseline (possibly due to extra precautions taken by cancer patients to reduce their exposure?).

We did not specifically mention cancer in the results because small inverse associations were seen for a number of outcomes in the comparison with influenza, so we did not think it justified to single out this outcome. We do make the more general point that … “Compared with influenza patients, people in the COVID-19 group had similar *or lower* risk of admission or death from most causes” (Results).

We agree that the lower risk of cancer outcomes specifically may have been driven in part by slightly lower baseline rates of cancer. However, it is also recognised that there was a reduction in health seeking for non-COVID conditions during the first months of the pandemic, which may have driven the lower risks of a wider range of outcomes that we observed. We have added this point to the discussion.

“This is supported by the more similar risks we observed when COVID-19 survivors were compared with people who had experienced influenza hospitalisation, with risks for some outcomes actually lower in the COVID-19 group, possibly linked to a general reduction in health seeking for non-COVID conditions in the early months of the pandemic[23].” (Discussion, findings in context)

Reviewer #4

SUMMARY: This cohort study of over 164,000 hospitalized adults (n=24,673 discharged following COVID-19 between discharged between 1st February and 30th December 2020; n=123,362 matched general population controls in 2019; and n=16,058 discharged following influenza between 2017-2019) compares the medium and long-term risks of hospital admission and death, overall and by specific cause across the three study groups. It uses administrative data sources from linked primary care and hospital data in the OpenSAFELY platform, which is reported to cover approximately 40% of the population in England. The main finding identifies that people discharged following hospitalization for COVID-19 had higher associated risks for rehospitalization and death than the general population, similar risks compared to those hospitalized for influenza.

The paper is well written and addresses an evolving, poorly studied, and important area of health policy and planning as it relates to the care of patients who survive hospitalization for COVID-19. Early data indicate these individuals appear to be at high risk for ongoing health needs and high resource use. The present study appears to build on a recent study in the UK (Ayoubkhani D et al BMJ 2021) by including an "active control" population of adults hospitalized with influenza and with a longer study follow-up. The study has clear applications to healthcare resource planning and policy in the care of these individuals suggesting a substantial extra burden on healthcare systems in the future.

I have a few concerns with the study as it stands that I believe should be addressed to improve the overall quality of this paper, which I hope are viewed as helpful.

4.1 The authors used Cox regression models to estimate hazard ratios for the defined outcomes. However, a key assumption of this modelling approach is that these HRs are constant over time. Prior literature on risk of death or rehospitalization demonstrates higher risk in the early period following discharge from hospital, as does Figure 1a provided by the authors. It would be helpful if the authors included time as an interaction term in their modelling to help delineate how these risks change over time, including the possibility of categorizing the time variable into clinically relevant post-discharge time frames (e.g. 30-, 90-, 180-day readmission).

Thank you for this excellent suggestion, which we have taken on board. We have added analysis investigating potential changes in hazard ratios over time, by fitting interactions between exposure and follow-up time as suggested. We detected that for some outcomes a more strongly raised risk early was indeed seen earlier in follow-up. We have reported this important extra insight in the paper.

**Changes**: “Interactions with follow-up time (classified as <30 days, 30-<90 days and ≥90 days from hospitalisation [COVID-19/influenza groups] or entry [general population controls]) were examined to investigate whether any increased risk was concentrated in early follow-up, and as an implicit check of proportional hazards.” (Methods, Statistical analysis)

“We found evidence of changes over time in the hazard ratios of several outcomes, with more pronounced raised risks earlier following COVID-19 hospitalisation (Appendix Figure A2).” (Results)

“Risks were higher for all categories of disease-specific hospital admissions/deaths after a COVID-19 hospitalisation than in general population controls, with excess risks more pronounced earlier in follow-up for several outcomes.” (Discussion, key findings)

Appendix Figure A2 added to show the results of models containing interaction between exposure and follow-up time.

4.2 Why did the authors choose not to adjust for admission to ICU in their modelling as a surrogate for disease severity? There were nearly 11% of COVID-19 patients and only 0.1% of influenza patients admitted to ICU. This may serve to substantially overestimate the adjusted risks of mortality among patients with COVID-19. Further focus on this could be added to the limitations in the discussion section as well.

We did not intend any assumption that COVID and influenza patients were equally sick. As the reviewer notes, a substantially higher proportion of COVID-19 patients were admitted to ICU, reflecting the now well-established higher risk of serious disease with SARS-Cov-2 compared with influenza infection. We still think the influenza comparison group is useful. First, as noted by the reviewer 1 (comment 1.3), this comparison group helps to account for the general raised risks associated with any hospital admission. Secondly, influenza is a related infectious disease and has been used as a benchmark for measuring COVID-19 risks and impacts since the start of the pandemic. We further expanded on the rationale for this comparison group in the introduction.

We do agree that it would be an interesting further exploration to look at how the results change when adjusted for critical care use, but a major caveat is that receipt of critical care is not a straightforward marker of disease severity. The patients who are most frail and/or not expected to survive may not be recommended for critical care, and in addition critical care beds in England were at times under pressure during the COVID-19 pandemic further complicating clinical decision-making, so results adjusted for critical care use are not entirely straightforward to interpret. Nevertheless, to explore whether the variable might have had a major impact on our findings, we have now added adjustment for critical care use in a sensitivity analysis; in fact this had little impact on the hazard ratios. We have reported this new sensitivity analysis in the paper.

**Change**: “We aimed to compare post-COVID risks with two separate comparison groups: (i) the general population, and (ii) people hospitalised for influenza prior to the current pandemic. The latter was included to provide a comparison with risks after hospitalisation in general, using admissions from a well-characterised infectious disease.” (Introduction)

“Further sensitivity analyses included … adjusting for receipt of critical care in hospital (COVID-19 versus influenza comparison only) …” (Methods, statistical analyses)

“Our results changed little in a range of sensitivity analyses, including … adjusting for … receipt of critical care in hospital … (Appendix Figure A3).” (Results)

“Our findings were robust in a range of sensitivity analyses.” (Discussion, strengths and limitations)

Figure A3 added which includes results of sensitivity analyses adding adjustment for critical care use.

4.3 Related to #2 above, why did the authors not adjust for readmission risk using one of the many available readmission risk indices (see Kansagara D et al JAMA 2011 for a comprehensive list)?

We adjusted for a wide range of factors, covering demographics, comorbidities and lifestyle factors, substantially overlapping with factors included in readmission risk indices. We felt that adjustment for individual variables was optimal, as estimates are then fitted to the data per variable, whereas a pre-defined index would incorporate fixed weighting developed for a different purpose. Adding an index to the model on top of overlapping component variables would risk collinearity and model instability. In addition, some variables included in readmission indices in the linked paper would not be relevant in this setting (e.g. insurance status) or were unavailable in our data source (e.g. marital status).

4.4 The cause-specific outcomes among adults with COVID-19 may be artificially higher than those with Influenza due to availability bias. Put simply, patients and providers may be much more aware of COVID-19 and its complications, including those related to return to hospital than might be the case for those with pneumonia or even confirmed influenza. This appears to be supported by the data presented in this study. For example, the authors report that, "A substantial proportion of deaths in the COVID-19 group had COVID-19 listed as the underlying cause (500/2022, 24.7%), while in the influenza group ≤5 deaths were coded with influenza as the underlying cause"; and "Compared with influenza patients, people in the COVID-19 group had similar or lower risk of admission or death from most causes, but higher risks of admission/death from COVID-19/influenza/lower respiratory tract infection (LRTI, adjusted HR 1.37, 1.22-1.54); in the post-COVID-19 group these outcomes were
dominated by codes for COVID-19 itself (515/1122 [46%] of hospitalizations and 342/368 [93%] of deaths)". I worry that the conclusions drawn from this study in relation to the influenza comparator group may be different when considering the lack of adjustment for disease severity and this potential source of bias.

We did not intend any assumption that COVID and influenza patients were equally sick. Please see our response to comment 4.2 for our rationale for including this comparison, and details of an additional sensitivity analysis adjusting for critical care use. We agree with the point that higher awareness may have encouraged coding of COVID-19 causes of death/hospitalisation in the COVID-19 group; we have added this point.

**Changes**:

(see also changes under response 4.2)

“It is possible that high levels of awareness of COVID-19 during the pandemic may have encouraged coding of subsequent deaths as COVID-19-related, leading to overestimation in the comparison with historical influenza hospitalisations.” (Discussion, findings in context)

4.5 It is unclear as to why the authors chose to report cumulative incidence of the outcomes at 6 months when the maximum duration of follow-up for COVID-19 patients is 315 days (and the general population group was censored using this time frame). Is this the median follow-up time for the cohort? Further clarification as to why this specific time point was chosen would be informative.

We present cumulative incidence visually across the range of follow-up in Figures 2 and 4; a single time-point was chosen to provide a numerical illustration of differences between groups for the text, and we chose 6 months as a “round number” time point that would be intuitive for the reader, and avoiding time points near the maximum follow-up when a substantial portion of the study population may have been censored, making estimates more uncertain. We have added a clarification in the Results text.

**Change**: “Cumulative incidence of subsequent hospital admission or death after study entry in the COVID-19 group was higher than in general population controls, but similar to that in the influenza group (cumulative incidence at 6 months [for illustration] = 34.8, 15.2 and 37.8% in the three groups respectively; fully adjusted HR across all follow-up =… cumulative incidence curves over all follow-up shown in Figure 2a …” (Results)

4.6 The authors compare baseline characteristics of the three study groups and report for example that, "The COVID-19 group were more likely to be obese, non-White and less likely to be current smokers than both comparison groups". It would be helpful to report standardized differences in Table 1 to compare the magnitude of differences between study groups, which may inform some of the residual confounding that may be unaccounted for.

Thank you for the suggestion. Our feeling is that Table 1 gives enough information to illustrate similarities/key differences between the three exposure groups, and that there would be limited insight from further quantification, which does not necessarily correlate with degree of confounding (since confounding depends on association with both exposure and outcome).

4.7 There is a risk of misclassification of hospitalization for COVID using ICD-10 code U07.2 "COVID-19 - virus not identified". To test the robustness of their findings, could the authors repeat a sensitivity analysis among those with only the ICD-10 code "U07.1 COVID-19 - virus identified"?

We agree that there is some risk of misclassification due to the inclusion of U07.2 codes, though most COVID-19 hospitalisations included did have the code U07.1 (confirmed infection). To provide extra reassurance, we have now added a sensitivity analyses restricting to these cases, and results were very similar.

**Changes**:
“Further sensitivity analyses included restricting the COVID-19 group to those with a confirmed infection ICD-10 code (U071)…” (Methods, statistical analyses)

“Our results changed little in a range of sensitivity analyses, including restricting the COVID-19 group to the 21,770/24,673 (88%) with confirmed infection …” (Results)

“Our findings were robust in a range of sensitivity analyses.” (Discussion, strengths and limitations)

Figure A3 added which includes results of sensitivity analyses restricting the COVID-19 group to those with code U07.1 (COVID-19 - virus identified).

4.8 I am concerned about the presence of immortal time bias in requiring that adults survive 7 days post discharge, which also serves to underestimate the magnitude of healthcare resource needs among adults who survive hospitalization for COVID-19. There were ~3,700 patients who died within 7 days of discharge or were transferred to hospice (where they presumably died), which is ~15% of the final cohort of adults hospitalized with COVID-19 (n=24,673). The authors should mention this in their limitations and modify the interpretation of the study to include that this applies to adults who survive at least 1 week following hospitalization for COVID-19.

We agree that it is important to emphasise in our interpretation that the study applied to one-week survivors and we have clarified this in key sections, and added a note on this generalisibility issue to the limitations. We are confident that there is no immortal time bias because the first week of follow-up was not included in the denominator (i.e. patients were only counted as “at risk” from day 8, see Methods), whereas immortal time bias generally results from counting time as “at risk” when the outcome event cannot possible occur.

**Changes**:

“…we conducted a cohort study, using linked primary care and hospital data in OpenSAFELY to compare risks … between people discharged from COVID-19 hospitalisation (February-December 2020) and surviving at least one week, and …” (Abstract, methods)

“Patients discharged from a COVID-19 hospitalisation and surviving at least a week had more than double the risk of subsequent hospitalisation or death…” (Discussion, key findings)

“COVID-19 patients in our study had to survive at least a week to enter the study, so our results will not capture the total public health burden from point of discharge given a substantial number of deaths and readmissions in the first week following discharge; however we felt that excluding this first week enabled a focus on medium and longer-term post-acute outcomes, and avoided our results being dominated by deaths and readmissions due to premature discharge and transfers to other hospitals.” (Discussion, strengths and limitations)

“Patients surviving a COVID-19 hospitalisation for at least a week after discharge were at substantially higher risk than the general population for a range of subsequent adverse outcomes over a period of up to 10 months’ follow-up included in this study.” (Discussion, conclusion)

4.8 How confident are the authors in the accuracy of hospital coding for influenza admissions? Are these hospital coding practices based on PCR confirmed influenza?

We only included patients where influenza was coded as the primary reason for hospitalisation, and we believe this is likely to have been confirmed by PCR in most cases as part of routine care for a case serious enough to require hospital admission. However, we have no data on this, and we have added this to the limitations.

**Change**: “We had no data on whether influenza hospitalisations were confirmed by PCR testing, raising the possibility of misclassification in this comparator, though we only included cases where influenza was coded as the primary reason for hospitalisation.” (Discussion, strengths and limitations)

4.9 Can the authors comment on the generalizability of the study in using the OpenSAFELY data? Specifically, there are considerations about the generalizability to the overall study cohort, and to those that acquired COVID. The low proportion of patients residing in care homes (4.9% in the COVID population and 1.3% in the matched general population) may suggest this is a healthier, less frail population overall.

Our data cover primary care practices that use one particular electronic health record software system (TPP SystmOne), which is used by approximately 40% of GPs across England. The sample is somewhat geographically clustered, due to popularity of different GP software systems in different regions, but is otherwise expected to be representative of the broader population, given that the vast majority of the population (estimated at around 98%) is registered with a GP. We have added a note on the geographic clustering to the discussion. We believe that the low proportion classified as being in care homes reflects a relatively low sensitivity of our care home classification (as now mentioned in the methods – please see also response to reviewer 2 comment 2.2); for this reason we only used this variable in descriptive and sensitivity analyses.

**Change**: “…our data source covered around 40% of the population of England, giving us high statistical power, though it should be noted that our study population would not have been geographically representative of England, since TPP SystmOne software is more widely used than other systems in parts of Eastern and Southern England and , and used less than other software in London.^27^” (Discussion, strengths and limitations)

4.10 I believe the paper could be improved with a more substantive discussion of the policy applications for health and human resource planning in the care of patients who survive hospitalization for COVID-19 beyond, "These findings suggest a need for services to support and closely monitor people following discharge from hospital with COVID-19." For example, how might these patients require more support? Will it require more frequent follow-up from their General Practitioners? Does the healthcare system need to provide education to healthcare providers to improve their comfort and competency to deal with the potentially unique needs of these patients? Should governments consider financial motivations to incentives the care of this vulnerable population?
Thank you, we have now expanded on this, taking on board the suggestions above.

**Change**: “These findings suggest a need for services to support and closely monitor people following discharge from hospital with COVID-19, for example through more frequent/active follow-up in primary care in the weeks and months following a hospitalisation. Our results can be used to help inform healthcare providers and raise awareness of potential complications during this period. Our findings will also help with public health resource planning in the context of high rates of SARS-CoV-2 infection in many countries. Ongoing monitoring will be important to investigate whether these patterns persist in the light of new variants and increasing levels of vaccination.” (Discussion, conclusion)
